# Tests of a New Drowsiness Characterization and Monitoring System Based on Ocular Parameters

**DOI:** 10.3390/ijerph13020174

**Published:** 2016-01-29

**Authors:** Clémentine François, Thomas Hoyoux, Thomas Langohr, Jérôme Wertz, Jacques G. Verly

**Affiliations:** 1 Laboratory for Signal and Image Exploitation, Department of Electrical Engineering and Computer Science, University of Liège, Liège 4000, Belgium; thomas.hoyoux@ulg.ac.be (T.H.); tlangohr@ulg.ac.be (T.L.); jacques.verly@ulg.ac.be (J.G.V.); 2 Phasya s.a. Company, Angleur 4031, Belgium; j.wertz@phasya.com

**Keywords:** drowsiness, monitoring, photooculography, polysomnography, psychomotor vigilance test, drowsy driving

## Abstract

Drowsiness is the intermediate state between wakefulness and sleep. It is characterized by impairments of performance, which can be very dangerous in many activities and can lead to catastrophic accidents in transportation or in industry. There is thus an obvious need for systems that are able to continuously, objectively, and automatically estimate the level of drowsiness of a person busy at a task. We have developed such a system, which is based on the physiological state of a person, and, more specifically, on the values of ocular parameters extracted from images of the eye (photooculography), and which produces a numerical level of drowsiness. In order to test our system, we compared the level of drowsiness determined by our system to two references: (1) the level of drowsiness obtained by analyzing polysomnographic signals; and (2) the performance of individuals in the accomplishment of a task. We carried out an experiment in which 24 participants were asked to perform several Psychomotor Vigilance Tests in different sleep conditions. The results show that the output of our system is well correlated with both references. We determined also the best drowsiness level threshold in order to warn individuals before they reach dangerous situations. Our system thus has significant potential for reliably quantifying the level of drowsiness of individuals accomplishing a task and, ultimately, for preventing drowsiness-related accidents.

## 1. Introduction

Drowsiness is the intermediate state between wakefulness and sleep. It is characterized by an uncontrollable desire to sleep and by impairments of performance, e.g., slow reaction time, loss of vigilance, and deficits in information processing [1,2]. The level of drowsiness of an individual often varies as a function of time. This variation can to a large extent be explained by the interaction between two processes, *i.e.*, the circadian rhythm and the homeostatic process [3]. The first is governed by our internal biological clock (resulting in high sleep pressure during the night and particularly the early morning hours), and the second represents the increase in sleep pressure with continuous hours of wakefulness [3]. However, an individual’s level of drowsiness can also be affected by many other factors, such as suffering from sleep disorders, taking medications or alcohol, executing very monotonous tasks, *etc.* [4,5]. The combination of the above processes and factors results in the fact that some individuals may be drowsy at any time during the day, and sometimes all day long.

The consequences of drowsiness may be catastrophic. Drowsiness is indeed involved in a large number of accidents, whether in transportation or in industry [6,7]. In the driving context for example, drowsiness leads to difficulties in maintaining a constant speed, a correct distance between vehicles, and a proper position on the road, resulting in an increase of the risk of road accidents. In a more general context, drowsiness impairs one’s judgment and ability to execute a task correctly [2]. Characterizing drowsiness, monitoring its level, and determining the times when it reaches a dangerous level thus constitute an important “grail” and valuable endeavor in public health and safety.

We have thus developed an innovative drowsiness characterization and monitoring system that is suitable for operational use and that continuously, objectively, and automatically produces a level drowsiness on a numerical scale. This system is based on several key ocular parameters, the values of which are obtained from images of the eye. Ocular parameters are indeed recognized to be excellent and reliable physiological indicators of drowsiness [8,9,10,11,12]. Moreover, the use of images of the eye, *i.e.*, photooculography (POG), appears to be the best method for characterizing drowsiness in operational settings since it is completely physiology-based, task independent, and non-invasive.

The primary goal of this study is to verify that the level of drowsiness produced by our POG-based drowsiness monitoring system is well related with two references: (1) the level of drowsiness obtained by analyzing polysomnographic signals; and (2) the performance of individuals in the accomplishment of Psychomotor Vigilance Tests (PVTs). Polysomnography (PSG), *i.e.*, the analysis of EEG, EOG, and EMG signals, is indeed considered to be the “Gold Standard” to study drowsiness, but it is very sensitive to artifacts, and it is not very practical for everyday use, in part because of the possible presence of electrodes [13,14]. The PVT is based on reaction times and is considered in the scientific literature as a reliable tool to assess drowsiness, but it involves a specific task (the PVT), which also constitutes a distraction from the main task of the person (such as driving), and is thus not suitable for all operational settings [15,16,17]. A secondary goal of this study is to determine the best threshold among our POG-based levels of drowsiness in order to alert individuals before they become dangerous.

## 2. Material and Method 

### 2.1. Data Acquisition

**Figure 1:**
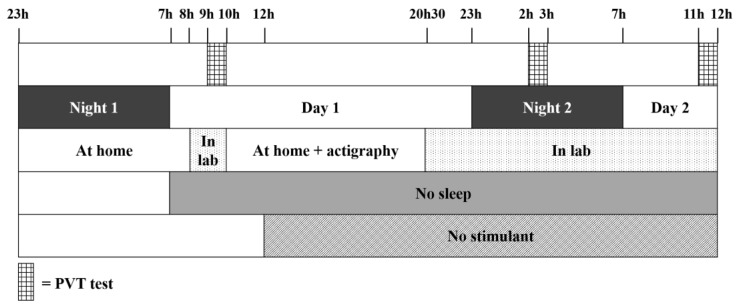
Data acquisition protocol. The first horizontal line represents the times when the participants had to perform a Psychomotor Vigilance Test (PVT). The second line refers to the succession of nights and days, and the third line shows when the participants were at home and in the lab. The two last lines indicate from what time the participants were sleep deprived and could no longer consume any stimulant.

Twenty-four healthy volunteers (11 M, 13 F, mean age 22.7, range 19–34 years) participated in the experiment, which included performing three Psychomotor Vigilance Tests (PVTs)—each of 10 min in duration—in different sleep conditions over two days. The selection of the volunteers was based on the following criteria: being between age from 18 to 35 years, no drug addiction, no sleep pathologies, not being a shift worker, and no jet lag during the two preceding weeks. For ease of explanation, the experiment can be viewed as the succession of Night 1, Day 1, Night 2, and Day 2, and as consisting of three PVTs (PVT 1, 2, and 3). Figure 1 provides an illustration of the protocol that we used. The participants provided a written, informed consent prior to the study.

On Night 1, the participants slept at home and were asked to report the number of hours of sleep using a sleep diary (the mean ± SD for all participants is 7.57 ± 0.8 h of sleep, range 6.5–9.0 h). Then, the participants were not allowed to sleep from the time they woke up on Day 1 until the end of the study (12:00 on Day 2). At 8:00 on Day 1, the participants arrived at our laboratory and took PVT 1, between 9:00 and 10:00. They were then free to leave the laboratory to carry out their normal activities but were equipped with an actigraph (Actiwatch 2, Philips Respironics) in order to check that they had not slept. The participants came back to our laboratory at 20:30 on Day 1. On Night 2, they took PVT 2 between 2:00 and 3:00 and, after breakfast on Day 2, they took PVT 3 between 11:00 and 12:00 (and after at least 28 h of sleep deprivation). At the end of the study, the participants were sent back home. They were asked not to consume any stimulant (coffee, tea, *etc*.) from noon on Day 1 until the end of PVT 3. This protocol was approved by the Ethics Committee of the University of Liège.

During each PVT, we recorded (1) images of the right eye; (2) polysomnographic (PSG) signals; and (3) data coming from the PVT. Before each test, the participants were also asked to evaluate their level of drowsiness using the Karolinska Sleepiness Scale (KSS) [13], but this data is not analyzed in this study.

### 2.2. POG-Based Drowsiness Monitoring

The right eye of each participant was monitored using a POG drowsiness monitoring system that we have developed (prototype of Drowsimeter R100, from Phasya). This system is in the form of a pair of eyeglasses on which there are a high-speed camera, an infrared illumination source, and a hot mirror. The system automatically provides the values of several ocular parameters extracted from images of the eye and that are linked to the movement of the eyelids (including blinks) and the movement of the eyeball (including saccades). The ocular parameters are defined, in this paper, over successive time windows of 20 s to be coherent with reference measures. Each ocular parameter thus has a numerical value per 20-s window. Here are some examples of ocular parameters that we extract from images of the eye:

- the mean duration of blinks [9,10,11];
- the PERCLOS 80 (which is the proportion of time in a 20-s window that the eye is at least 80% closed) [8];
- the percentage of microsleeps (with a microsleep being defined here as an eye closure of at least 0.5 s) [9].

We thus obtain the values of a set of several ocular parameters for each 20-s window of interest. From this set of ocular parameter values, we can derive a score that represents the state of wakefulness/drowsiness of the individual, which we call a POG-based level of drowsiness. This POG-based level of drowsiness, produced automatically by our system, is a numerical value between 0 (well awake) and 10 (very drowsy). 

### 2.3. KDS-Based Drowsiness Monitoring

The brain activity and the ocular activity of each participant were also recorded and analyzed using the Karolinska Drowsiness Scale (KDS) [13,14]. The KDS was developed by Åkerstedt *et al.* to help determine the state of wakefulness or drowsiness of a person in “active” situations (operational settings) at a given time. In this particular scale, the electroencephalogram (EEG) is used to detect the presence of alpha (8–12 Hz) activity and/or theta (4–8 Hz) activity, and the electrooculogram (EOG) is used to detect slow eye movements (SEMs). The KDS scoring method is based on Rechtschaffen and Kales’ (1968) scoring rules [18], and consists in visually determining a KDS score for each successive window of 20 s based on the presence of signs of drowsiness, *i.e.*, alpha activity and/or theta activity in the EEG, and/or SEMs in the EOG. The procedure involves dividing a 20-s window in 10 sub-windows of 2 s each. If one detects the presence of at least one sign of drowsiness in any sub-window, the score for the window is incremented by 1. The KDS score determined for each 20-s window is thus a numerical value between 0 (well awake) and 10 (very drowsy). In our study, a trained scorer analyzed the data and assigned a KDS score to each 20-s window, which we call a KDS-based level of drowsiness.

### 2.4. Performance 

The performance of each participant in the accomplishment of a task was assessed using the PVT. The PVT is indeed a performance task that estimates the ability of individuals to sustain attention and to respond to visual stimuli in an environment conducive to sleep. The PVT is based on the assumption that drowsiness increases the reaction time and the rate of false response [15,16,17].

The PVT used in our study is our own implementation of the PVT defined by Dinges [15,16,17]. Each participant was instructed to monitor a computer screen presenting a red rectangular box and to press a button as quickly as possible after detecting a yellow millisecond counter appearing inside the rectangle. Upon pressing the button, the reaction time was displayed for 1 s, giving feedback to the subject. The inter-stimulus interval varied randomly from 2 to 10 s. The millisecond counter timed out after 30 s without any response. Each test lasted 10 min.

The data recorded for each stimulus are the time when the counter starts, $t_{s}$, and the time when the subject responds, $t_{r}$. The reaction time (RT) is defined as $RT=t_{r}-t_{s}$, where $t_{r}$ corresponds to the first response after at least 100 ms from the start of the counter. If the subject responds with a delay of at least 500 ms from the start of the counter, we report the result as a “lapse”. For each 20-s window, we determined the following outcomes: the mean RT (including lapses) and the percentage of lapses. The percentage of lapses is defined here as the ratio between the number of lapses and the number of stimuli over the 20-s time window.

## 3. Results

### 3.1. Effect of Sleep Deprivation 

We computed the mean POG-based level of drowsiness, the mean KDS-based level of drowsiness, the mean reaction time (RT), and the mean percentage of lapses for all participants for the three sleep conditions (three PVTs). The results are presented in Table 1.

**Table 1:** Mean levels of drowsiness (photooculography (POG) and Karolinska Drowsiness Scale (KDS)), mean reaction time (RT), and mean percentage of lapses for all participants for the three PVTs.

Measure	PVT 1	PVT 2	PVT 3
*Mean POG-based level of drowsiness + SD*	2.48±1.37	3.63±1.91	4.81±2.21
*Mean KDS-based level of drowsiness + SD*	0.77±1.29	1.25±1.80	2.26±1.99
*Mean RT (ms) + SD*	417±260	453±251	535±540
*Mean percentage of lapses (%) + SD*	10.10±21.84	15.57±25.83	25.74±30.30

We also performed separate repeated ANOVA measurements to distinguish the effects of sleep deprivation on the levels of drowsiness and the (levels of) performance of the participants. The results are presented in Table 2. 

**Table 2:** Results of the separate, repeated ANOVA measurements (one degree of freedom) to distinguish differences in the levels of drowsiness (POG and KDS) and the (levels of) performance (RT and percentage of lapses) of all participants from PVT 1 to PVT 2 and from PVT 2 to PVT 3. Significant effects are shown in boldface.

Measure	From PVT 1 to PVT 2		From PVT 2 to PVT 3	
	F	pval	F	pval
Mean POG-based level of drowsiness	95.89	<0.01	139.55	<0.01
Mean KDS-based level of drowsiness	7.18	<0.01	127.64	<0.01
Mean RT (ms)	0.37	0.54	21.85	<0.01
Mean percentage of lapses (%)	1.12	0.29	53.38	<0.01

From Table 1 and Table 2, we observe the following:

- Mean POG-based level of drowsiness: a significant increase from PVT 1 to PVT 2, and from PVT 2 to PVT 3.
- Mean KDS-based level of drowsiness: a significant increase from PVT 1 to PVT 2, and from PVT 2 to PVT 3.
- Mean RT: no significant increase from PVT 1 to PVT 2, but a significant increase from PVT 2 to PVT 3.
- Mean percentage of lapses: no significant increase from PVT 1 to PVT 2, but a significant increase from PVT 2 to PVT3.

### 3.2. Relations between the POG-Based Level of Drowsiness and Both References

For each 20-s window of test, we computed the POG-based level of drowsiness, the KDS-based level of drowsiness (reference), the mean RT (reference), and the percentage of lapses (reference). We can thus associate each reference value to a POG-based level of drowsiness and compute the mean of all reference values for each POG-based level of drowsiness. Below, we place all values of the POG-based level of drowsiness in successive bins of integer values (from 0 to 10).

**Figure 2:**
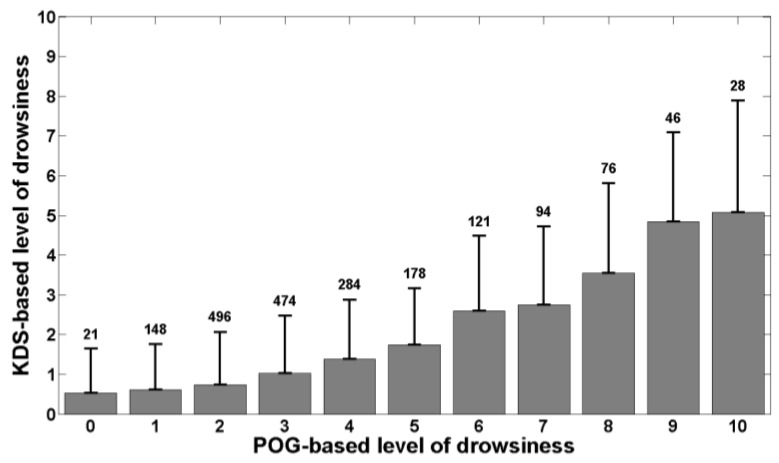
KDS-based level of drowsiness as a function of POG-based level of drowsiness. Each bin represents the mean KDS-based level of drowsiness for each value of POG-based level of drowsiness. The error bars show the standard deviation around each mean value and the number above each bin indicates the number of 20-s windows falling into the bin.

**Figure 3:**
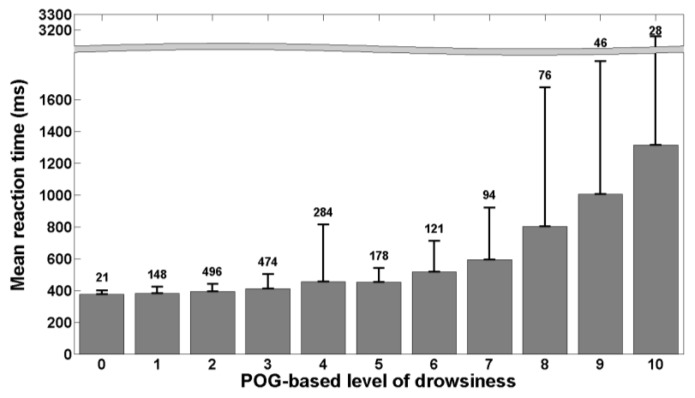
Mean reaction time as a function of POG-based level of drowsiness. Each bin represents the mean reaction time for each value of POG-based level of drowsiness. The error bars show the standard deviation around each mean value and the number above each bin indicates the number of 20-s windows falling into the bin.

**Figure 4:**
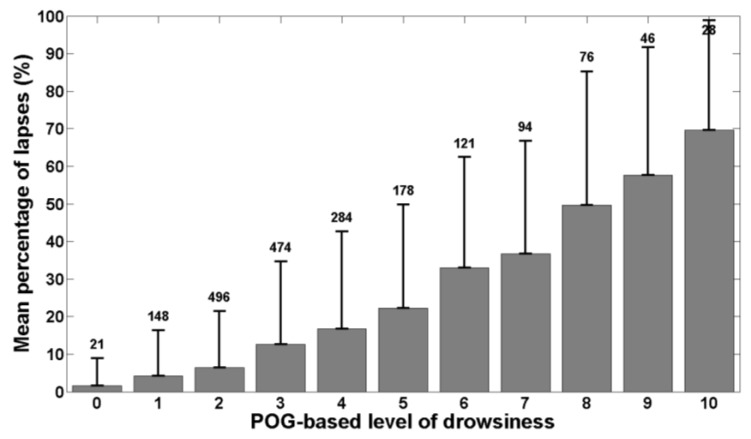
Mean percentage of lapses as a function of POG-based level of drowsiness. Each bin represents the mean percentage of lapses for each value of POG-based level of drowsiness. The error bars show the standard deviation around each mean value and the number above each bin indicates the number of 20-s windows falling into the bin.

Figure 2 shows the mean KDS-based level of drowsiness and its standard deviation for each bin of POG-based level of drowsiness. The number above each bin represents the number of 20-s windows falling into the bin. Figure 3 is similar to Figure 2 and shows the mean RT and its standard deviation for each bin of POG-based level of drowsiness. Figure 4 shows the mean percentage of lapses and its standard deviation for each bin of POG-based level of drowsiness.

Correlation coefficients were also computed between our POG-based level of drowsiness and the references. The Pearson’s correlation coefficient between POG-based levels of drowsiness and KDS-based levels of drowsiness is positive and high (R=0.54, pval<0.01). The same finding is obtained for the correlation coefficient between the POG-based level of drowsiness and the percentage of lapses (R=0.52, pval<0.01). However, the Pearson’s correlation coefficient between the POG-based level of drowsiness and the mean reaction time is positive but weak (R=0.35, pval<0.01).

### 3.3. POG-Based Level of Drowsiness as Predictor of Lapses 

**Figure 5:**
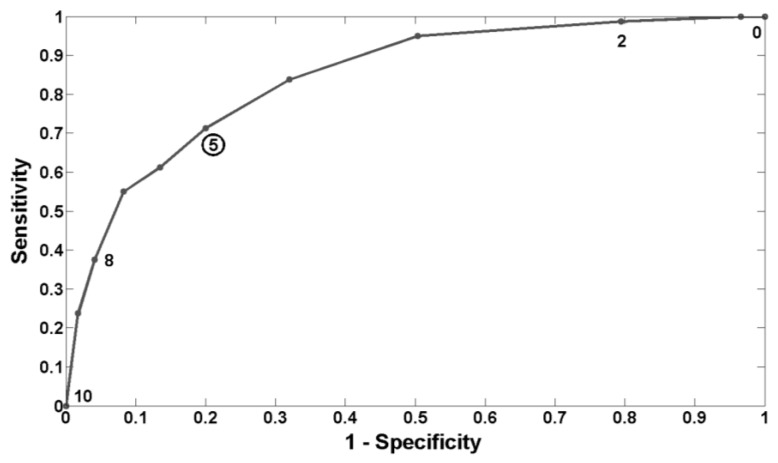
ROC curve with “lapsing” as parameter. Each dot on the ROC curve represents a threshold of POG-based level of drowsiness (0–10). The dot with the circled value (5) is the best compromise between sensitivity and specificity.

In order to determine the best threshold among the POG-based levels of drowsiness to alert individuals before they become dangerous, we used the concept of the common Receiver Operating Characteristic (ROC) curve. Since we consider that lapsing during the PVT is synonymous with being dangerous while performing critical tasks like driving a vehicle, we decided to find the best threshold among our POG-based levels of drowsiness that would best predict lapses. To do this, we took integer thresholds from 0 to 10 (representing the different values of drowsiness levels that we can obtain) and we computed values of sensitivity and specificity based on confusion tables for each threshold. The best threshold is the one with the highest probability of correct detection but with the lowest probability of false detection, *i.e.*, the best compromise between sensitivity and specificity. A threshold of 5 on our POG-based scale of drowsiness seems to be the best to predict lapses, and this threshold corresponds to a sensitivity of 72% and to a specificity of 80%. Figure 5 shows the ROC curve, and the point corresponding to the best threshold is highlighted with a circle.

## 4. Discussion

We have presented a new and innovative POG-based drowsiness monitoring system that automatically and continuously produces a level of drowsiness. We have shown in our experiments that the POG-based level of drowsiness produced by our system increased significantly with sleep deprivation on average for all participants. This result is perfectly in line with previous studies that have shown a rise in drowsiness with an increase in sleep deprivation [19], but the difference here is that the level of drowsiness is determined fully automatically via an objective drowsiness monitoring system. We also verified that the same behavior was found for the three reference measures used to validate our system (KDS-based levels of drowsiness, the mean RT, and the percentage of lapses). We indeed observed a significant augmentation of the KDS-based levels of drowsiness with sleep deprivation although the mean values of the KDS-based level of drowsiness during the PVT 2 and PVT 3 seem low compared to the maximum value they can take (10). This can be explained by the fact that low KDS-based levels of drowsiness are already found to be associated with performance decrements. Anund and colleagues indeed showed in one of their studies that there was already a significant change in the variability of the lateral position of the vehicle on the road for KDS ≥ 2 [20].

Another observation resulting from our experiments is that the standard deviation increased with sleep deprivation for all four measures above (POG-based level of drowsiness, KDS-based level of drowsiness, mean RT, and percentage of lapses). This means that there are much more differences between subjects in the sleep-deprived condition than in the not-sleep-deprived condition. Some individuals are indeed more affected by drowsiness than others. This greater level of variability in drowsiness and performance during sleep deprivation was also found by Doran *et al.* in 2001 [21]. One potential explanation is that different individuals have different chronotypes; some people are indeed more “evening” than “morning”, and they thus better support drowsiness at night [22], but this has to be investigated in more detail in a further study.

The correlations we obtained also show that our POG-based level of drowsiness is well related with both references. Indeed, Figure 2 shows that the KDS-based level of drowsiness increases with the POG-based level of drowsiness produced by the system we have developed and this is confirmed by Pearson’s correlation coefficient. As polysomnography is the physiological reference, this means that our system correctly assesses the actual, physiological state of drowsiness of individuals. Figure 3 and Figure 4 show that the mean reaction time and the percentage of lapses increase also with the POG-based level of drowsiness determined by our system. The computed correlation coefficients reveal that the POG-based level of drowsiness is more correlated with the percentage of lapses than with the mean reaction time. This indicates that our POG-based level of drowsiness is well correlated with performance impairments during the execution of a task. These results demonstrate the relevance of the level of drowsiness determined by our POG-based drowsiness monitoring system. However, this validation was performed in laboratory conditions. A further study should also evaluate this new, end-to-end POG-based monitoring system in real conditions, e.g., while driving.

In Section 3.3, we found that a threshold of 5 on our scale of drowsiness (from 0 to 10) would be the best to predict lapses. Of course, it should be clear that this threshold is the best compromise between sensitivity and specificity for the dataset considered in this study. Moreover, depending on the application, one may want to be more “sensitive” than “specific”, or conversely. In the case of critical tasks like driving for example, being less sensitive may lead to catastrophic consequences and it may be better to sound an alarm more often to maximize correct detections. However, too many false alarms are not desirable either, as the operator may decide to ignore them.

Compared to some other existing drowsiness monitoring systems that are based on the behavior of the person or on the behavior of the process in which he/she is involved (e.g., driving a car) [23], and that can be influenced by several factors (e.g., climatic conditions, road conditions, *etc.*), our POG-based drowsiness monitoring system has the advantage of objectively quantifying the real physiological level of drowsiness of the person and of being task independent. Our system is indeed based on a combination of several ocular parameters to produce a level of drowsiness and it is not related to the task performed by the person being monitored. It can therefore be used in many different applications. Moreover, compared to other technologies that use ocular parameters [24], the use of images of the eye enables a more precise measurement of the level of drowsiness. The behavior of the eyes is indeed a time-space phenomenon so the best way to characterize it and to isolate specific eye movements is to use images.

## 5. Conclusion 

The system based on images of the eye that we have developed is a promising tool to characterize drowsiness, monitor its level, and determine the times when it reaches a dangerous level. We have indeed demonstrated that the POG-based level of drowsiness produced by our system is well correlated with the KDS-based level of drowsiness and with impairments of performance while performing a PVT. We have also determined that the best drowsiness level threshold to predict lapses is 5 on the scale from 0 to 10. Our system also has the advantage of being non-invasive, usable in any condition, and of requiring no intervention from the person/user. It thus has significant potential for reliably quantifying the level of drowsiness of individuals accomplishing a task, and, ultimately, for preventing drowsiness-related accidents.
